# *miR-146a* C/G polymorphism increased the risk of head and neck cancer, but overall cancer risk: an analysis of 89 studies

**DOI:** 10.1042/BSR20171342

**Published:** 2018-01-10

**Authors:** Dezhong Sun, Xiaoyan Zhang, Xiaolei Zhang

**Affiliations:** 1 Department of Otolaryngology, Linyi City People’s Hospital, Linyi 276000, China; 2 Department of Oncology, Linyi City People’s Hospital, Linyi 276000, China; 3 Department of Geratology, Linyi City People’s Hospital, Linyi 276000, China

**Keywords:** cancer risk, head and neck cancer, meta-analysis, miR-146a C/G, polymorphism

## Abstract

Several studies have evaluated the association of *miR-146a* C/G with head and neck cancer (HNC) susceptibility, and overall cancer risk, but with inconclusive outcomes. To drive a more precise estimation, we carried out this meta-analysis. The literature was searched from MEDLINE (mainly PubMed), Embase, the Cochrane Library, and Google Scholar databases to identify eligible studies. A total of 89 studies were included. The results showed that *miR-146a* C/G was significantly associated with increased HNC risk in dominant model (*I*^2^ =15.6%, *P*_heterogeneity_=0.282, odds ratio (OR) =1.088, 95% confidence interval (CI) =1.002–1.182, *P*=0.044). However, no cancer risk was detected under all genetic models. By further stratified analysis, we found that rs4919510 mutation contributed to the risk of HNC amongst Asians under homozygote model (*I*^2^ =0, *P*_heterogeneity_=0.541, OR =1.189, 95% CI =1.025–1.378, *P*=0.022), and dominant model (*I*^2^ =0, *P*_heterogeneity_=0.959, OR =1.155, 95% CI =1.016–1.312, *P*=0.028). Simultaneously, in the stratified analysis by source of controls, a significantly increased cancer risk amongst population-based studies was found under homozygote model, dominant model, recessive model, and allele comparison model. However, no significant association was found in the stratified analysis by ethnicity and source of control. The results indicated that *miR-146a* C/G polymorphism may contribute to the increased HNC susceptibility and could be a promising target to forecast cancer risk for clinical practice. However, no significant association was found in subgroup analysis by ethnicity and source of control. To further confirm these results, well-designed large-scale case–control studies are needed in the future.

## Introduction

Cancer, although an age old disease, still poses a formidable challenge to researchers and clinicians. Little is known about its initiation, sustenance, progression and metastasis, and resistance and remission. Due to its morbidity and mortality, cancer is one of the most dreaded diseases and the related fatalities are majorly attributed to delayed diagnosis and treatment. Head and neck cancer (HNC), the sixth most frequent kind of cancer worldwide, is a group of biologically similar cancers that originate from head and neck regions such as oral cavity, pharyngeal cavity, and larynx [1]. Multifactors such as smoking, drinking, betel quid chewing, papilloma virus infection, and exposure to toxic substances are suggested to be the etiological risk factors for HNC [2,3]. Nevertheless, though many individuals are exposed to these external factors, HNC develops only in a small proportion of the exposed people, indicating that intrinsic factors such as genetic polymorphism might play critical roles in its carcinogenic mechanisms.

miRNAs represent a class of evolutionarily conserved, endogenous, single-stranded, non-coding RNA molecules of ~20 nts that regulate gene expression by degrading mRNAs or suppressing translation. miRNAs have been implicated in a wide range of physiologic and pathologic processes, including development, cell differentiation, proliferation, apoptosis, and carcinogenesis [4,5]. Accumulating evidence indicates that the expression of roughly 10–30% of all human genes is regulated by miRNAs [6]. More than half of the known miRNAs are located in cancer-associated genomic regions, and miRNAs are thought to contribute to oncogenesis because they can function either as tumor suppressors or oncogenes [7]. Analyses in human epithelial malignancies have shown that cancers can be distinguished and classified by distinct tumor-specific miRNA signatures [8]. Some of the key dysregulated miRNAs could serve as molecular biomarkers, leading to improved diagnosis and monitoring of cancer treatment response [9–11].

Single nucleotide polymorphisms (SNPs) are a type of common genetic variations associated with population diversity, disease susceptibility, drug metabolism, and genome evolution [12]. SNPs may affect the expression and function of miRNAs, which could therefore contribute to the susceptibility to cancer occurrence and development [13–16]. *miR-146a* C/G is located in the stem region opposite to the mature *miR-146a* sequence, which is suspected to have an effect on tumor immune responses and ultimately the development of cancer. In recent years, the polymorphism rs2910164 in *miR-146a* has attracted wide attention and many studies have been published to explore the association between SNPs of miRNAs and susceptibility to various cancers. But the results were not conclusive and consistent. Since SNPs in miRNAs are closely associated with head and neck cancer (HNC) susceptibility, it is necessary to assess whether these SNP polymorphisms are the risk factors for HNC. It is reported that meta-analysis is a well-established method for combining all the results from the available published information to produce a single estimate for quantitating gene–disease associations more precisely to increase the statistical power [17]. Thus, we performed this meta-analysis of case–control studies to estimate the importance of pre-*miR-146a* C/G polymorphism for HNC susceptibility.

## Materials and methods

### Publication search

A comprehensive electronic search was performed to identify articles published up until 12 November 2016 in MEDLINE (mainly PubMed), Embase, the Cochrane Library, and Google Scholar using the following search terms: ‘*miR-146a*’ or ‘rs2910164’ and ‘head and neck cancer’ or ‘cancer’ or ‘tumor’ or ‘carcinoma’ and ‘polymorphism’ or ‘SNPs’ or ‘variation’. All eligible studies published in English were retrieved, and their bibliographies were checked for additional relevant publications. Review articles and bibliographies of other identified relevant studies were searched by hand to identify any additional eligible studies.

### Inclusion and exclusion criteria

Studies included in this meta-analysis had to meet all of the following criteria: (i) case–control study evaluating the association between *miR-146a* C/G polymorphism and susceptibility to HNC and overall cancer; (ii) sufficient published data for calculating odds ratios (ORs) with corresponding 95% confidence intervals (CIs); (iii) full-text manuscript; and (iv) only the most recent or complete study reporting on the same population of patients was included. Exclusion criteria included: (i) reviews, other meta-analyses, comments, letters, and editorial articles; (ii) not a case–control study; and (iii) no usable data reported.

### Data extraction

Information regarding the following aspects was independently retrieved from each study by two reviewers: the first author’s surname, year of publication, country of origin, ethnicity, study design, total number of cases and controls, source of cases and controls, detected sample, genotyping methods, allele and genotype frequencies of cases and controls, and evidence of Hardy–Weinberg equilibrium (HWE) in the controls. In studies including subjects of more than one ethnicity, genotype data were extracted separately for each ethnic group. Data from one publication may contain more than one seperate case-control studies. Any discrepancies between the reviewers were resolved through discussion to reach a consensus.

### Statistical analysis

We used crude ORs with 95% CIs to explore the association between *miR-146a* C/G polymorphism and the risk of HNC and overall cancer. Five genetic variation models were analyzed: homozygote model (CC compared with GG), heterogeneity model (GC compared with CC), dominant model (CC + GC compared with GG), recessive model (CC compared with GC + GG), and allele comparison model (C compared with G). *P*-value of HWE in control group of each study was calculated by *χ*^2^ test and *P*<0.05 presented a state of disequilibrium [18]. We also performed subgroup analyses by ethnicity and source of control, and heterogeneity was calculated by *χ*^2^-based Q-statistic [19]. Both random-effects model (when *P*-value of heterogeneity was less than 0.05) and fixed-effects model (when *P*-value of heterogeneity was more than 0.05) were used [20,21]. Sensitivity analyses were performed to verify if our present results were stable. Begg’s funnel plots and Egger’s linear regression tests were used to examine possible publication bias [22,23]. All statistical analyses were performed using Stata software version 11.0 (StataCorp LP, College Station, TX, U.S.A.). All statistical analyses were two-sided, and *P*-values <0.05 were considered statistically significant.

## Results

### Characteristics of eligible studies

**Figure 1:**
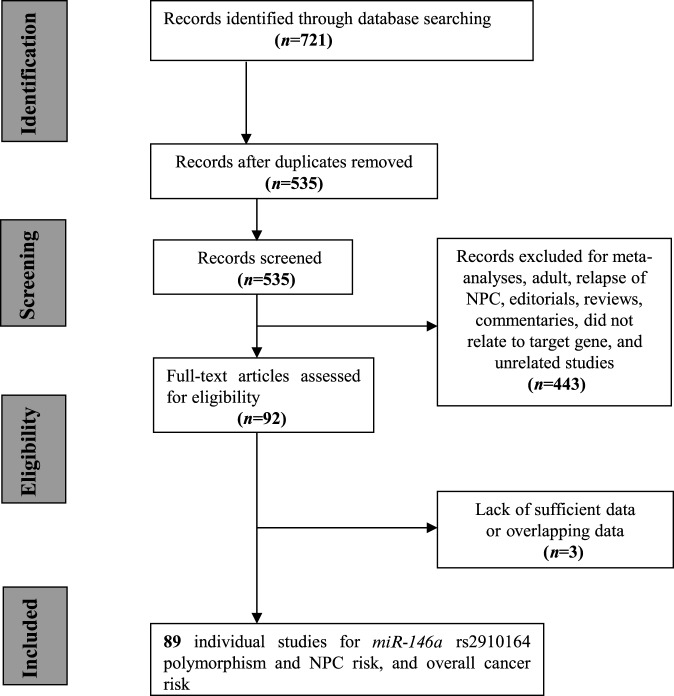
The process of literature research

A total of 721 articles were retrieved after the first search in PubMed, Embase, the Cochrane Library, and Google Scholar. Selection following the specified criteria eliminated 632 studies, leaving 89 individual studies [24–103]. The details of the selection process are presented in Figure 1. The publication years of included articles ranged from 2008 to 2016. The distributions of *miR-146a* C/G genotype in all studies were in accordance with HWE in the control group. No significant differences were found between cases and controls with respect to gender and age distributions. The modified quality scores of all studies ranged from 9 to 16, with 71% (5/7) of the included studies classified as high quality (≥12).The characteristics of all included studies are summarized in Table 1.

**Table 1:** Characteristics of all eligible studies

Reference	Year	Country	Ethnicity	Cancer type	Control source	Genotyping method	Sample size		Case			Control		
							Cases	Controls	GG	GC	CC	GG	GC	CC
Horikawa et al. [24]	2008	U.S.A.	Caucasian	Renal cell cancer	PB	SNPlex assay	261	235	144	103	14	126	94	15
Jazdzewski et al.^1^ [25]	2008	Finland	Caucasian	PTC	PB	SNPlex assay	206	274	99	104	3	150	105	19
Jazdzewski et al.^2^ [25]	2008	Poland	Caucasian	PTC	PB	SNPlex assay	201	475	115	82	4	286	163	26
Jazdzewski et al.^3^ [25]	2008	U.S.A.	Caucasian	PTC	PB	SNPlex assay	201	152	91	101	9	90	52	10
Xu et al. [26]	2008	China	Asian	Liver cancer	HB	PCR-RFLP	479	504	80	241	158	58	249	197
Yang et al. [27]	2008	U.S.A.	Caucasian	Bladder cancer	PB	SNPlex assay	691	674	414	242	35	385	258	31
Hoffman et al. [28]	2009	U.S.A.	Caucasian	Breast cancer	PB	massARRAY	439	478	234	176	29	273	178	27
Hu et al. [29]	2009	China	Asian	Breast cancer	HB	PCR-RFLP	1009	1093	165	515	329	180	551	362
Tian et al. [30]	2009	China	Asian	Lung cancer	PB	PCR-RFLP	1058	1035	360	510	188	364	502	169
Catucci et al.^1^ [31]	2010	Italy	Caucasian	Breast cancer	HB	Sequencing	754	1243	409	286	59	650	520	73
Catucci et al.^2^ [31]	2010	Germany	Caucasian	Breast cancer	HB	Sequencing	805	904	451	304	50	536	318	50
Guo et al. [32]	2010	China	Caucasian	ESCC	PB	SNaPshot	444	468	234	190	20	206	220	42
Liu et al. [33]	2010	U.S.A.	Mixed	SCCHN	HB	PCR-RFLP	1109	1130	630	411	68	655	405	70
Okubo et al. [34]	2010	Japan	Asian	Gastric cancer	HB	PCR-RFLP	552	697	73	243	236	121	322	254
Pastrello et al. [35]	2010	Italy	Caucasian	Breast and ovarian cancer	PB	Sequencing	101	155	60	36	5	90	59	6
Srivastava et al. [36]	2010	India	Asian	Gall bladder cancer	PB	PCR-RFLP	230	224	129	90	11	138	81	5
Xu et al. [37]	2010	China	Asian	Prostate cancer	HB	PCR-RFLP	251	280	68	135	48	54	150	76
Zeng et al. [38]	2010	China	Asian	Gastric cancer	HB	PCR-RFLP	304	304	62	153	89	53	132	119
Akkiz et al. [39]	2011	Turkey	Caucasian	Liver cancer	HB	PCR-RFLP	222	222	137	75	10	144	67	11
Garcia et al. [40]	2011	French	Caucasian	Breast cancer	PB	TaqMan	1130	596	676	388	66	352	220	24
George et al. [41]	2011	India	Asian	Prostate cancer	PB	PCR-RFLP	159	230	4	79	76	7	107	116
Hishida et al. [42]	2011	Japan	Asian	Gastric cancer	HB	PCR-RFLP	583	1637	82	271	230	229	775	633
Mittal et al. [43]	2011	India	Asian	Bladder cancer	PB	PCR-RFLP	212	250	127	79	6	135	108	7
Permuth-Wey et al. [44]	2011	U.S.A.	Caucasian	Glioma	PB	GoldenGate	593	614	345	198	50	375	214	25
Vinci et al. [45]	2011	Italy	Caucasian	NSCLC	PB	HRMA	101	129	44	48	9	73	45	11
Yue et al. [46]	2011	China	Asian	Cervical cancer	HB	PCR-RFLP	447	443	118	224	105	87	206	150
Zhang et al. [47]	2011	China	Asian	Liver cancer	HB	PIRA-PCR	925	1593	156	450	319	291	725	577
Zhou et al. [48]	2011	China	Asian	CSCC	HB	PCR-RFLP	226	309	43	113	70	34	159	116
Ma et al. [49]	2012	China	Asian	Gastric cancer	HB	Sequencing	86	42	20	44	14	6	19	14
Alshatwi et al. [50]	2012	Saudi	Asian	Breast cancer	PB	TaqMan	100	100	2	50	48	3	46	51
Chu et al. [51]	2012	China	Asian	Oral cancer	HB	PCR-RFLP	470	425	54	242	174	54	196	175
Hezova et al. [52]	2012	Czech	Caucasian	Colorectal	HB	TaqMan	197	212	115	70	12	124	79	9
Kim et al. [53]	2012	Korea	Asian	Liver cancer	PB	PCR-RFLP	286	201	27	159	100	24	103	74
Lung et al. [54]	2012	China	Asian	Nasopharyngeal carcinoma	PB	Tm-shift	229	3631	24	88	117	497	1721	1413
Mihalache et al. [55]	2012	Italy and Germany	Caucasian	Cholangiocarcinoma	HB	TaqMan	182	350	118	53	11	211	122	17
Min et al. [56]	2012	Korea	Asian	Colorectal	HB	PCR-RFLP	446	502	62	233	151	69	245	188
Wang et al. [57]	2012	China	Asian	Bladder cancer	HB	TaqMan	1017	1179	369	456	192	340	571	268
Xiang et al. [58]	2012	China	Asian	Liver cancer	HB	PCR-RFLP	100	200	27	45	28	45	100	55
Zhou et al. [59]	2012	China	Asian	Liver cancer	PB	PCR-RFLP	186	483	33	86	67	71	254	158
Zhou et al. [60]	2012	China	Asian	Gastric cancer	HB	TaqMan	1686	1895	578	822	286	551	951	393
Lv et al. [61]	2013	China	Asian	Colorectal cancer	PB	PCR-RFLP	353	540	54	230	47	96	274	143
Chae et al. [62]	2013	Korea	Asian	Colorectal cancer	PB	PCR-RFLP	399	568	61	182	156	121	282	165
Ma et al. [63]	2013	China	Asian	TNBC	HB	massARRAY	192	191	35	94	63	34	93	64
Ma et al. [64]	2013	China	Asian	Colorectal cancer	HB	TaqMan	1147	1203	444	534	169	397	614	192
Orsos et al. [65]	2013	Hungary	Caucasian	SCCHN	PB	PCR-RFLP	468	468	284	168	16	323	136	9
Vinci et al. [66]	2013	Italy	Caucasian	Colorectal cancer	PB	HRMA	160	178	86	57	17	100	65	13
Wei et al. [67]	2013	China	Asian	PTC	PB	massARRAY	753	760	136	323	294	138	345	277
Wei et al. [68]	2013	China	Asian	ESCC	HB	massARRAY	368	370	67	184	117	67	181	122
Yamashita et al. [69]	2013	Japan	Asian	Melanoma	PB	PCR-RFLP	50	107	0	35	15	3	53	51
Zhang et al. [70]	2013	China	Asian	HCC	PB	MassARRAY	997	998	163	503	331	156	475	367
Ahn et al. [71]	2013	Korea	Asian	Gastric cancer	HB	PCR-RFLP	461	447	71	231	159	62	221	164
Song et al. [72]	2013	China	Asian	Gastric cancer	HB	PCR-RFLP	1208	1166	199	586	423	207	615	344
Wu [73]	2014	China	Asian	Colorectal cancer	HB	ASA	175	300	22	59	80	53	120	114
Chu et al. [74]	2014	China	Asian	HCC	HB	PCR-RFLP	188	337	22	82	84	50	145	141
Cong et al. [75]	2014	China	Asian	HCC	HB	PCR-RFLP	206	218	27	85	94	17	84	117
Dikeakos et al. [76]	2014	Greece	Caucasian	Gastric cancer	HB	PCR-RFLP	163	480	13	45	105	24	149	307
Du et al. [77]	2014	China	Asian	Renal	HB	TaqMan assay	353	362	68	167	118	57	190	115
Hu et al. [78]	2014	China	Asian	Colorectal	HB	PCR-RFLP	200	373	34	82	84	44	187	142
Huang et al. [79]	2014	China	Asian	Nasopharyngeal	HB	PCR-RFLP	160	200	23	73	64	36	110	54
Jeon et al. [80]	2014	Korea	Asian	Lung	PB	PCR-RFLP	1091	1096	223	500	368	244	540	312
Jia et al. [81]	2014	China	Asian	NSCLC	HB	PCR-RFLP	400	400	64	182	154	76	200	124
Kupcinskas et al. [82]	2014	Germany, Lithuania, Latvia	Caucasian	Gastric	HB	TaqMan assay	362	347	252	94	16	223	108	16
Kupcinskas et al. [83]	2014	Lithuania, Latvia	Caucasian	Colorectal	HB	TaqMan assay	192	424	140	50	2	275	134	15
Mao et al. [84]	2014	China	Asian	Colorectal	PB	SNPscan system	547	561	70	291	186	85	271	205
Nikolić et al. [85]	2014	Serbia	Caucasian	Prostate	HB	TaqMan assay	286	199	184	90	12	129	63	7
Palmieri et al.^1^ [86]	2014	Italy	Caucasian	OSCC	HB	TaqMan assay	337	88	197	121	19	50	31	7
Palmieri et al.^2^ [86]	2014	Italy	Caucasian	OSCC	HB	TaqMan assay	337	206	197	121	19	105	84	17
Palmieri et al.^3^ [86]	2014	Italy	Caucasian	OSCC	HB	TaqMan assay	337	543	197	121	19	297	206	40
Parlayan et al.^1^ [87]	2014	Japan	Asian	Gastric	HB	TaqMan assay	160	524	20	79	61	71	237	216
Parlayan et al.^2^ [87]	2014	Japan	Asian	Lung	HB	TaqMan assay	148	524	25	67	56	71	237	216
Parlayan et al.^3^ [87]	2014	Japan	Asian	Prostate	HB	TaqMan assay	89	524	11	41	37	71	237	216
Pu et al. [88]	2014	China	Asian	Gastric	HB	PCR-RFLP	197	513	36	96	65	96	274	143
Qu et al. [89]	2014	China	Asian	ESCC	HB	Allele-specific PCR	381	426	62	203	116	75	228	123
Dikaiakos et al. [90]	2015	Greece	Caucasian	Colorectal	HB	PCR-RFLP	157	299	8	48	101	21	120	158
Gomez-Lira et al. [91]	2015	Italy	Caucasian	Melanoma	HB	PCR-RFLP	224	264	107	100	17	149	105	10
Qi et al. [92]	2015	China	Asian	Breast cancer	PB	PCR-RFLP	321	290	146	132	43	126	144	20
Zhu et al. [93]	2015	China	Asian	ESCC	HB	PCR-RFLP	248	300	82	120	36	99	139	40
Deng et al. [94]	2015	China	Asian	Bladder cancer	HB	PCR-RFLP	159	258	26	73	60	32	154	112
Li et al. [95]	2015	China	Asian	HCC	HB	PCR-RFLP	266	266	151	86	29	166	81	19
Shen et al. [96]	2015	China	Asian	ESCC	HB	SNaPshot multiplex system	1400	2185	220	685	495	345	1060	780
Yan et al. [97]	2015	China	Asian	HCC	HB	PCR-RFLP	274	328	35	145	94	36	169	123
Yin et al. [98]	2015	China	Asian	Lung cancer	HB	PCR-RFLP	575	608	97	280	198	127	313	168
Xia et al. [99]	2015	China	Asian	Gastric cancer	HB	TaqMan assay	1125	1196	192	536	397	199	577	420
Hashemi et al. [100]	2016	Iran	Caucasian	Prostate cancer	HB	T-ARMS-PCR assay	169	182	25	131	13	24	147	11
Jiang et al. [101]	2016	China	Asian	Gastric cancer	HB	MassARRAY	898	992	154	441	303	207	457	325
Miao et al. [102]	2016	China	Asian	HNSCC	HB	Illumina Infinium1 human exome BeadChip	576	1552	497	773	278	154	228	80
Chen et al.^1^ [103]	2016	Taiwan	Asian	OSCC	HB	TaqMan assay	512	668	71	241	200	103	293	272
Chen et al.^2^ [103]	2016	Taiwan	Asian	PSCC	HB	TaqMan assay	146	668	16	77	53	103	293	272
Chen et al.^3^ [103]	2016	Taiwan	Asian	OPSCC	HB	TaqMan assay	658	668	87	318	253	103	293	272

Abbreviations: BC, breast cancer; CRC, colorectal cancer; GC, gastric cancer; ESCC,esophageal squamous cell carcinoma; HB, hospital-based; HCC, hepatocellular carcinoma; HNSCC, squamous cell carcinoma of the head and neck; HRMA, high resolution melting analysis; LC, lung cancer; NSCLC, non-small-cell lung carcinoma; OPSCC, squamous cell carcinoma of the oral cavity, oropharynx, and hypopharynx; OSCC, oral squamous cell carcinoma; PB, population-based; *P*_hwe_, *P*-value of HWE; PSCC, squamous cell carcinoma of the oropharynx and hypopharynx; PTC, papillary thyroid cancer; RFLP, restriction fragment length polymorphism; SCCHN, squamous cell carcinoma of head and neck; TNBC, triple negative breast cancer.

^1,2,3^The superscript values 1, 2 and 3, indicate the number of studies (one, two and three respectively) covered the published article.

### *miR-146a* C/G polymorphism and HNC risk

**Figure 2:**
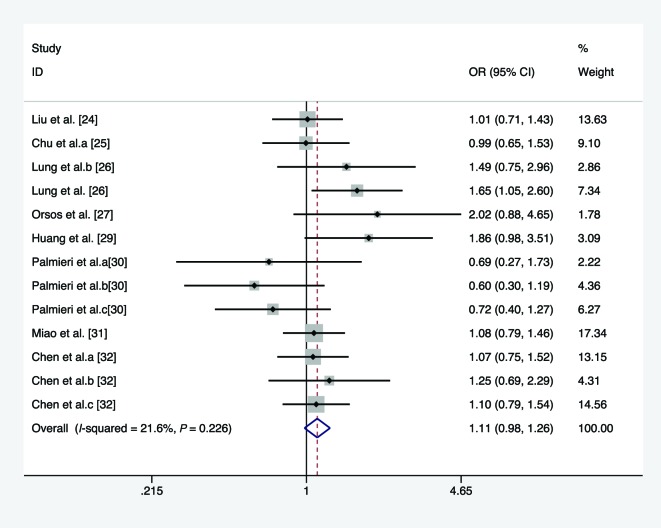
Forest plot of the association between *miR-146a* rs2910164 polymorphism and HNC risk (under homozygote model)

**Figure 3:**
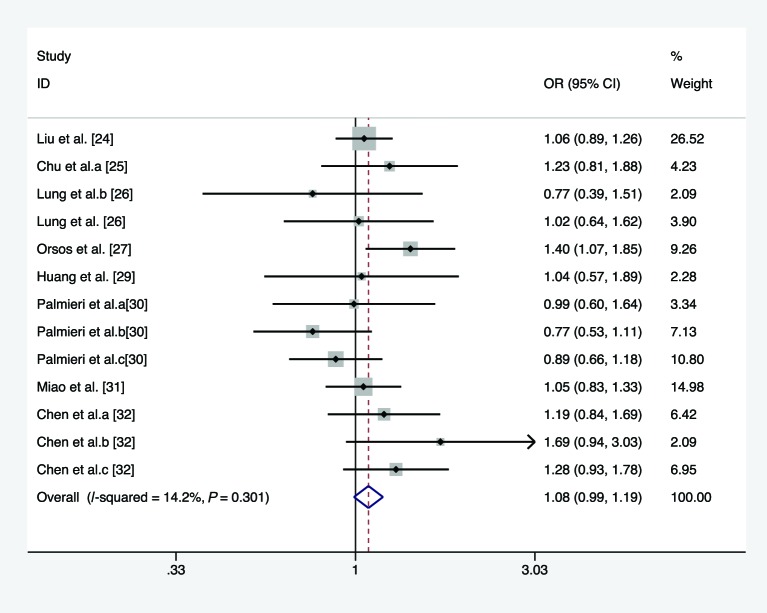
Forest plot of the association between *miR-146a* rs2910164 polymorphism and HNC risk (under heterozygote model)

**Figure 4:**
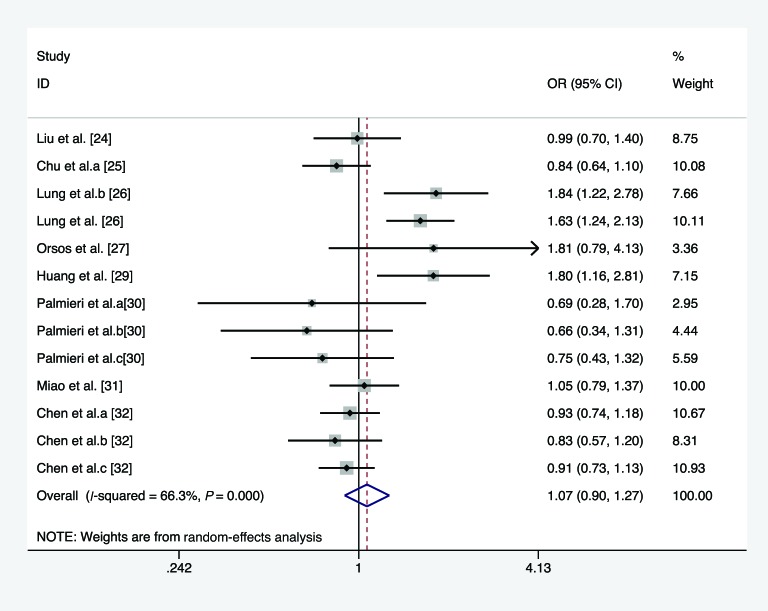
Forest plot of the association between *miR-146a* rs2910164 polymorphism and HNC risk (under recessive model)

**Figure 5:**
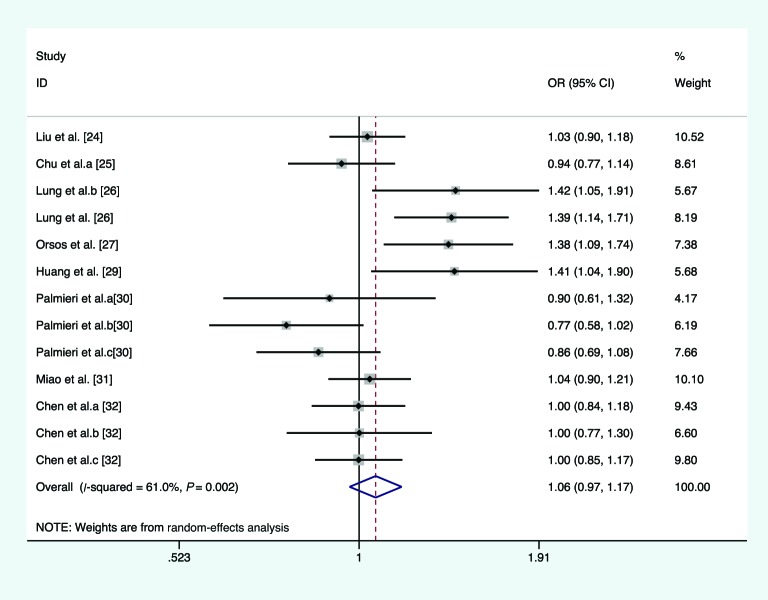
Forest plot of the association between *miR-146a* rs2910164 polymorphism and HNC risk (under allele comparison model)

**Figure 6:**
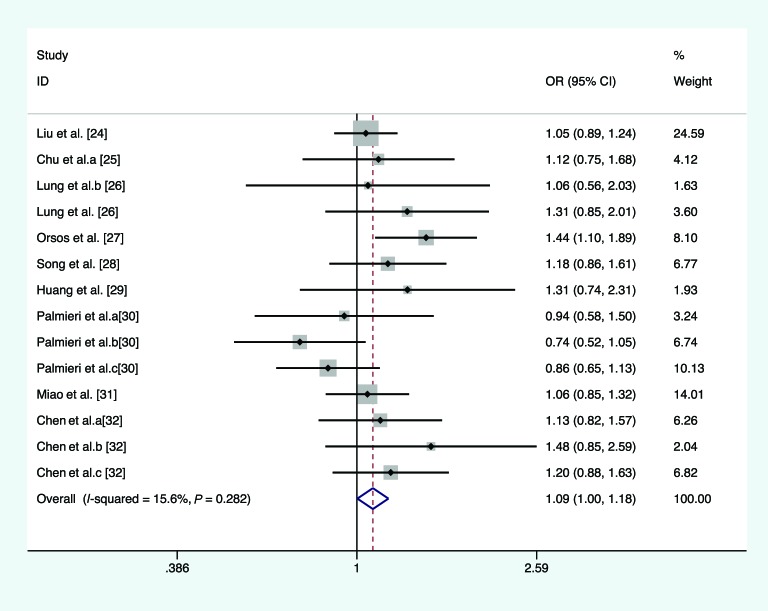
Forest plot of the association between *miR-146a* rs2910164 polymorphism and HNC risk (under dominant model**)**

In the overall analysis, we pooled 13 separate studies to explore the association between *miR-146a* C/G polymorphism and the risk of HNC under homozygote, heterozygote, recessive, and allele comparison model. There is no significant association between *miR-146a* C/G polymorphism and the risk of HNC under homozygote model (*I*^2^ =21.6%, *P*_heterogeneity_=0.226, OR =1.113, 95% CI =0.980–1.263, *P*=0.099, Figure 2), heterozygote model (*I*^2^ =14.2%, *P*_heterogeneity_=0.301, OR =1.084, 95% CI =0.991–1.186, *P*=0.079, Figure 3), recessive model (*I*^2^ =66.3%, *P*_heterogeneity_<0.01, OR =1.068, 95% CI =0.896–1.272, *P*=0.465, Figure 4), and allele comparison model (*I*^2^ =61%, *P*_heterogeneity_=0.002, OR =1.061, 95% CI =0.966–1.166, *P*=0.214, Figure 5). Furthermore, we pooled all 14 eligible studies to explore the association between pre-*miR-146a* C/G polymorphism and the risk of HNC. Significant association was found under dominant model (*I*^2^ =15.6%, *P*_heterogeneity_=0.282, OR =1.088, 95% CI =1.002–1.182, *P*=0.044, Figure 6). In the subgroup analysis by ethnicity, no significant association was found amongst Caucasians under homozygote model (*I*^2^ =36.7%, *P*_heterogeneity_=0.177, OR =0.919, 95% CI =0.716–1.180, *P*=0.509, Table 2), heterozygote model (*I*^2^ =52.7%, *P*_heterogeneity_=0.076, OR =1.040, 95% CI =0.922–1.173, *P*=0.521, Table 2), dominant model (*I*^2^ =58.6%, *P*_heterogeneity_=0.034, OR =1.027, 95% CI =0.857–1.232, *P*=0.772, Table 2), recessive model (*I*^2^ =10.9%, *P*_heterogeneity_=0.344, OR =0.919, 95% CI =0.719–1.174, *P*=0.449, Table 2), and allele comparison model (*I*^2^ =69%, *P*_heterogeneity_=0.012, OR =0.981, 95% CI =0.814–1.183, *P*=0.843, Table 2). Simultaneously, no associations were detected amongst Asians under heterozygote model (*I*^2^ =0, *P*_heterogeneity_=0.713, OR =1.142, 95% CI =0.997–1.308, *P*=0.054, Table 2), recessive model (*I*^2^ =76.5, *P*_heterogeneity_<0.01, OR =1.133, 95% CI =0.914–1.404, *P*=0.254, Table 2), and allele comparison model (*I*^2^ =57.6, *P*_heterogeneity_=0.021, OR =1.103, 95% CI =0.988–1.233, *P*=0.082, Table 2), while slight association was found amongst Asians under homozygote model (*I*^2^ =0, *P*_heterogeneity_=0.541, OR =1.189, 95% CI =1.025–1.378, *P*=0.022, Table 2) and dominant model (*I*^2^ =0, *P*_heterogeneity_=0.959, OR =1.155, 95% CI =1.016–1.312, *P*=0.028, Table 2). In the stratified analysis by source of controls, a significantly increased cancer risk amongst population-based studies was found under homozygote model (*I*^2^ =0, *P*_heterogeneity_=0.855, OR =1.668, 95% CI =1.183–2.352, *P*=0.004, Table 2), dominant model (*I*^2^ =0, *P*_heterogeneity_=0.674, OR =1.359, 95% CI =1.095–1.687, *P*=0.005, Table 2), recessive model (*I*^2^ =0, *P*_heterogeneity_=0.874, OR =1.697, 95% CI =1.367–2.107, *P*<0.001, Table 2), and allele comparison model (*I*^2^ =0, *P*_heterogeneity_=0.991, OR =1.394, 95% CI =1.215–1.599, *P*<0.001, Table 2), while no association was found amongst population-based studies under heterozygote model (*I*^2^ =3.5%, *P*_heterogeneity_=0.408, OR =1.219, 95% CI =0.974–1.526, *P*=0.083, Table 2). Meanwhile, no significant association was found amongst hospital-based studies under homozygote model (*I*^2^ =0, *P*_heterogeneity_=0.471, OR =1.113, 95% CI =0.980–1.263, *P*=0.603, Table 2), heterozygote model (*I*^2^ =40.5%, *P*_heterogeneity_=0.186, OR =1.060, 95% CI =0.961–1.169, *P*=0.248, Table 2), dominant model (*I*^2^ =0, *P*_heterogeneity_=0.462, OR =1.047, 95% CI =0.957–1.144, *P*=0.318, Table 2), recessive model (*I*^2^ =26%, *P*_heterogeneity_=0.204, OR =0.941, 95% CI =0.849–1.043, *P*=0.247, Table 2), and allele comparison model (*I*^2^ =19.8%, *P*_heterogeneity_=0.261, OR =0.994, 95% CI =0.935–1.056, *P*=0.837, Table 2). Results of the meta-analyses are presented in Table 2.

**Table 2:** Meta-analysis on the association between *miR-146a* rs2910164 polymorphism and HNC risk

Variables	Study number	Statistic model	Test of heterogeneity		Test of association		Publication bias	
			*P*	*I*^2^	OR (95% CI)	*P*	*P*_Begg’s_	*P*_Egger’s_
**Homozygote model**								
***Total***	13	Fixed	0.226	21.6	1.113 (0.980–1.263)	0.099	1.000	0.793
***Ethnicity***								
Caucasian	5	Fixed	0.177	36.7	0.919 (0.716–1.180)	0.509		
Asian	8	Fixed	0.541	0	1.189 (1.025–1.378)	**0.022**		
***Source of control***								
Population-based study	3	Fixed	0.855	0	1.668 (1.183–2.352)	**0.004**		
Hospital-based study	10	Fixed	0.471	0	1.113 (0.980–1.263)	0.603		
**Heterozygote model**								
***Total***	13	Fixed	0.301	14.2	1.084 (0.991–1.186)	0.079	0.855	0.968
***Ethnicity***								
Caucasian	5	Fixed	0.076	52.7	1.040 (0.922–1.173)	0.521		
Asian	8	Fixed	0.713	0	1.142 (0.997–1.308)	0.054		
***Source of control***								
Population-based study	3	Fixed	0.408	3.5	1.219 (0.974–1.526)	0.083		
Hospital-based study	10	Fixed	0.186	40.5	1.060 (0.961–1.169)	0.248		
**Dominant model**								
***Total***	14	Fixed	0.282	15.6	1.088 (1.002–1.182)	**0.044**	0.661	0.549
***Ethnicity***								
Caucasian	6	Random	0.034	58.6	1.027 (0.857–1.232)	0.772		
Asian	8	Fixed	0.959	0	1.155 (1.016–1.312)	**0.028**		
***Source of control***								
Population-based study		Fixed	0.674	0	1.359 (1.095–1.687)	**0.005**		
Hospital-based study		Fixed	0.462	0	1.047 (0.957–1.144)	0.318		
**Recessive model**								
***Total***	13	Random	<0.01	66.3	1.068 (0.896–1.272)	0.465	0.76	0.784
***Ethnicity***								
Caucasian	5	Fixed	0.344	10.9	0.919 (0.719–1.174)	0.449		
Asian	8	Random	<0.01	76.5	1.133 (0.914–1.404)	0.254		
***Source of control***								
Population-based study	3	Fixed	0.874	0	1.697 (1.367–2.107)	**<0.001**		
Hospital-based study	10	Fixed	0.204	26	0.941 (0.849–1.043)	0.247		
**Allele comparison model**								
***Total***	13	Random	0.002	61	1.061 (0.966–1.166)	0.214	0.855	0.587
***Ethnicity***								
Caucasian	5	Random	0.012	69	0.981 (0.814–1.183)	0.843		
Asian	8	Random	0.021	57.6	1.103 (0.988–1.233)	0.082		
***Source of control***								
Population-based study	3	Fixed	0.991	0	1.394 (1.215–1.599)	**<0.001**		
Hospital-based study	10	Fixed	0.261	19.8	0.994 (0.935–1.056)	0.837		

Values of *P*<0.05 were considered statistically significant.

### *miR-146a* C/G polymorphism and overall cancer risk

**Figure 7:**
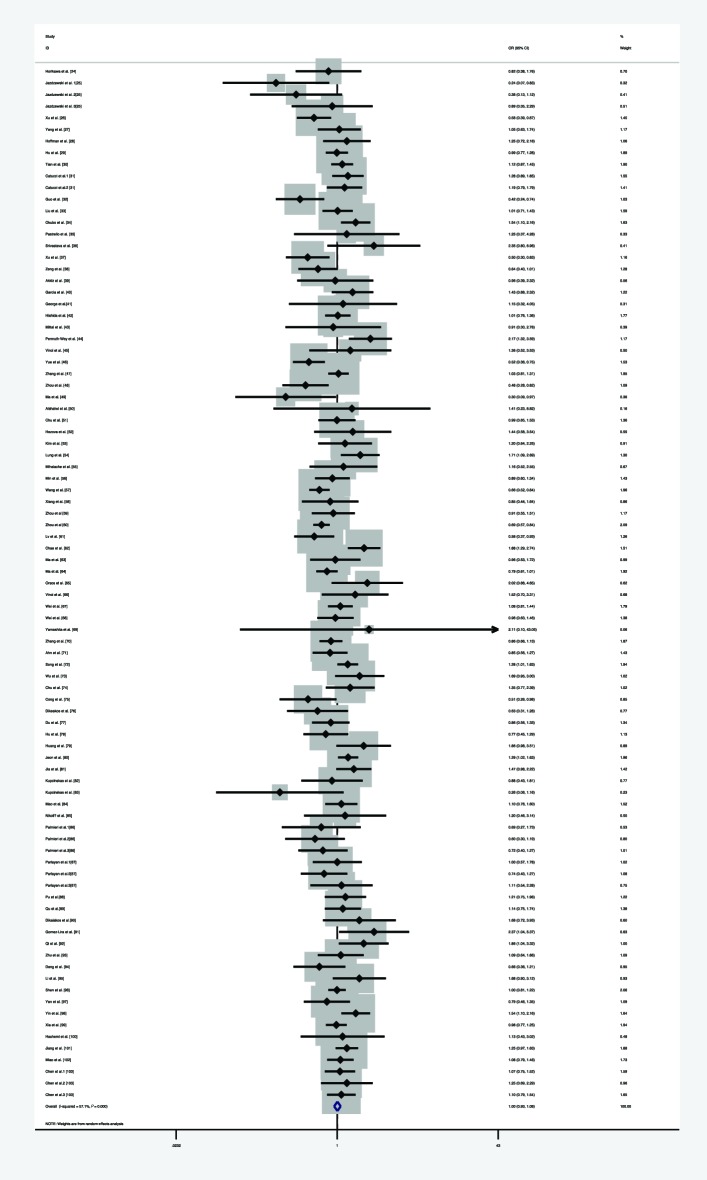
Forest plot of the association between *miR-146a* rs2910164 polymorphism and overall risk (under homozygote model)

**Figure 8:**
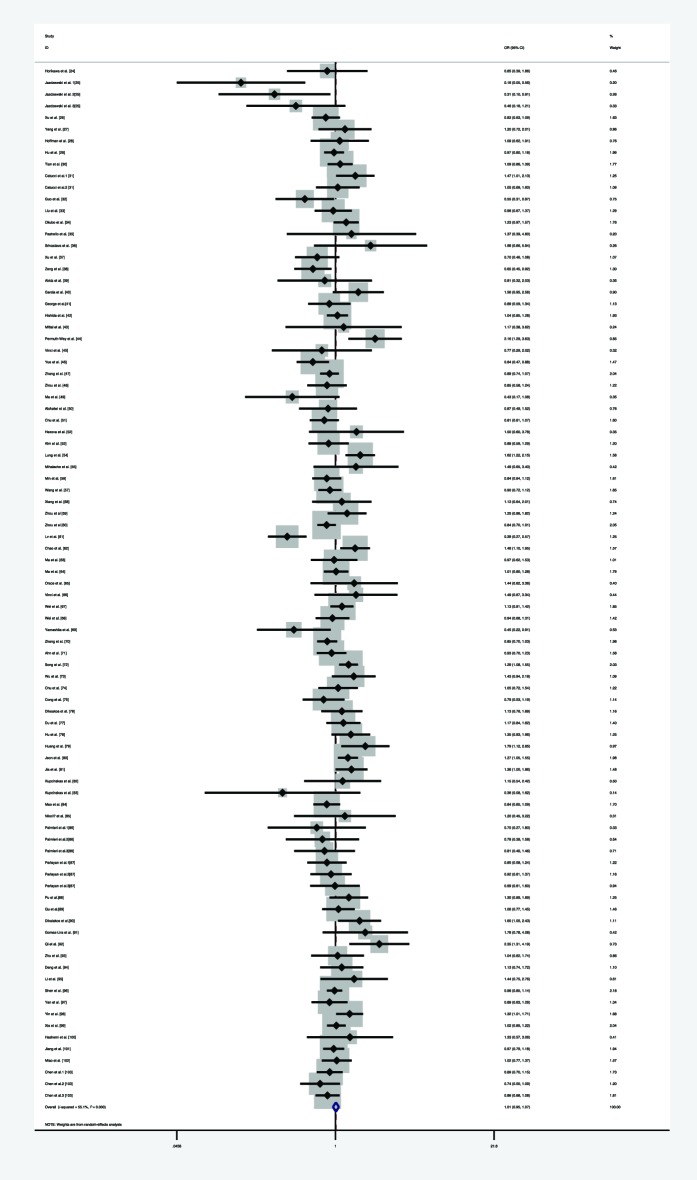
Forest plot of the association between *miR-146a* rs2910164 polymorphism and overall cancer risk (under heterozygote model)

**Figure 9:**
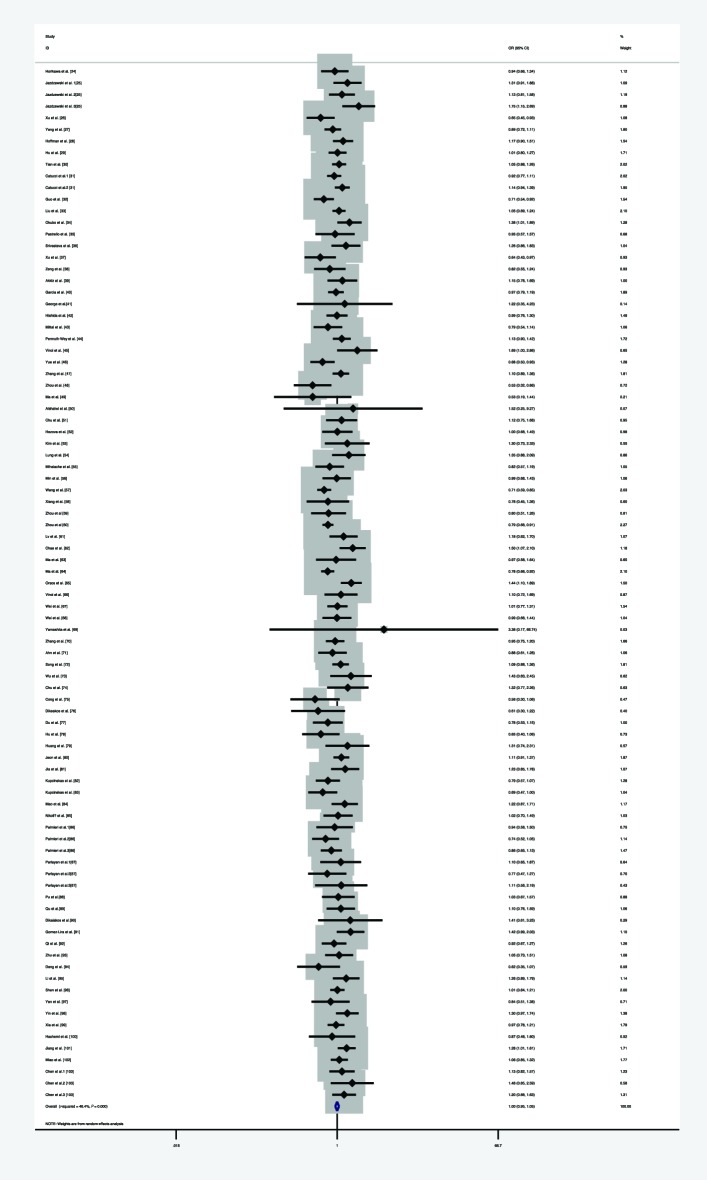
Forest plot of the association between *miR-146a* rs2910164 polymorphism and HNC risk (under dominant model)

**Figure 10:**
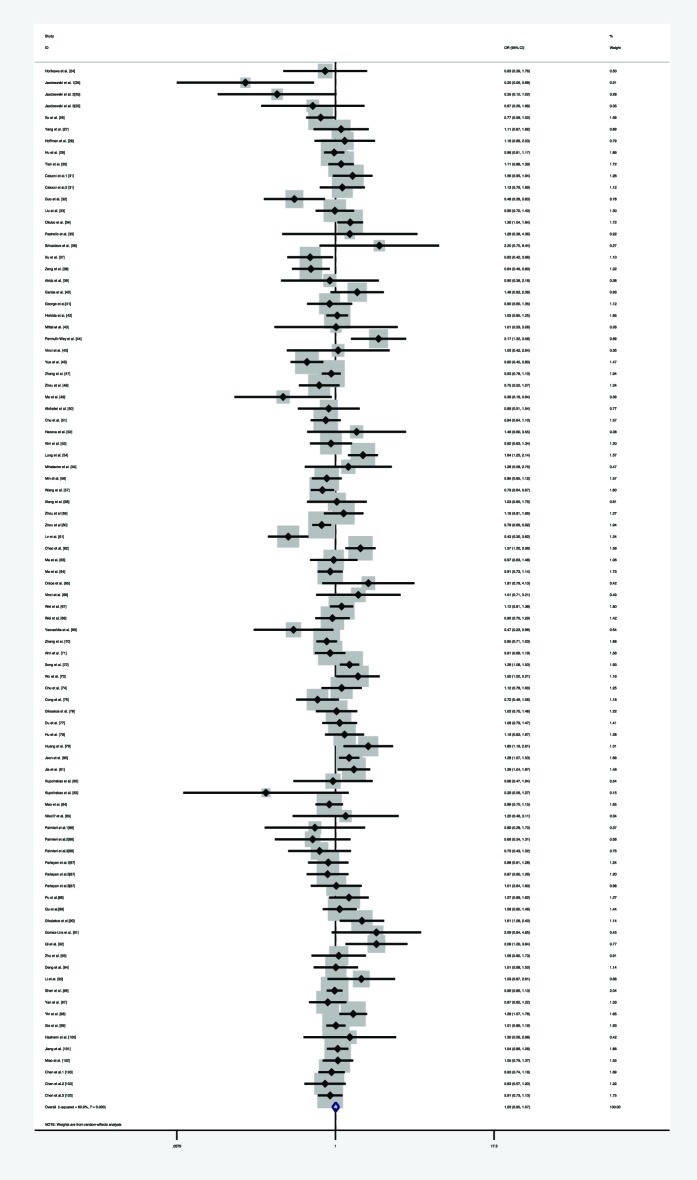
Forest plot of the association between *miR-146a* rs2910164 polymorphism and overall cancer risk (under recessive model)

**Figure 11:**
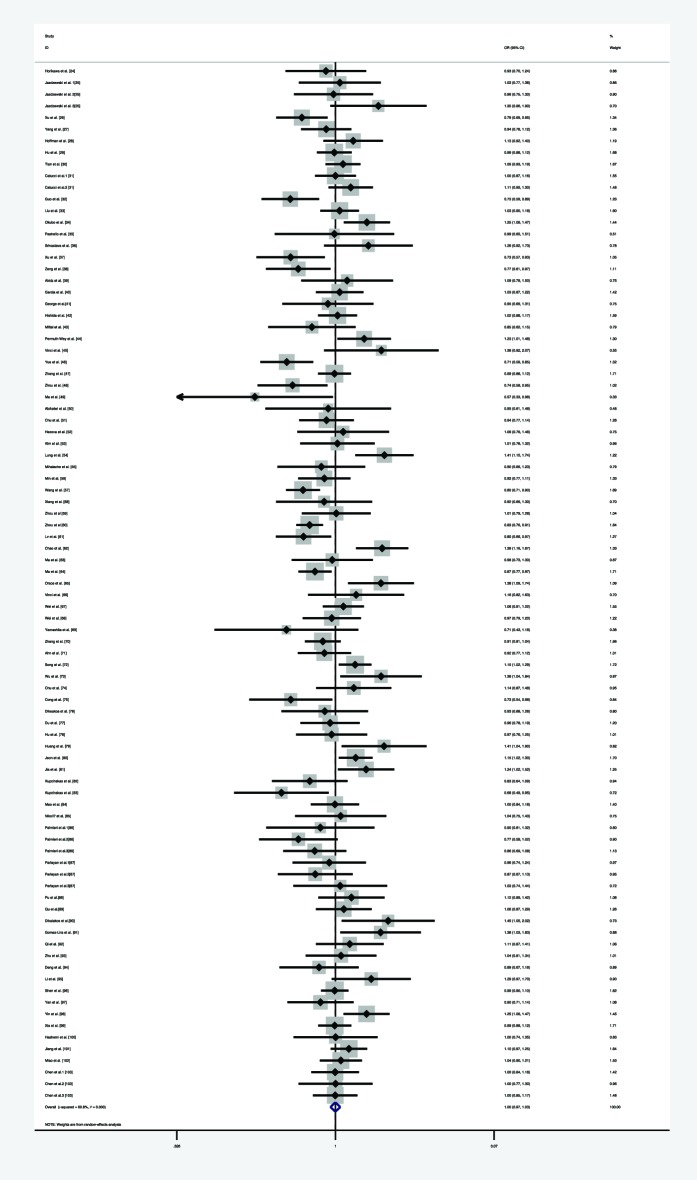
Forest plot of the association between *miR-146a* rs2910164 polymorphism and overall cancer risk (under allele comparison model)

Furthermore, we explored the association between the pre-*miR-146a* C/G polymorphism and overall cancer risk. We first analyzed the heterogeneity by Q-test and *I*-squared in any of the genetic models. Significant statistical heterogeneity was identified in the homozygote model (*I*^2^ =57.1%, *P*_heterogneity_<0.001), heterozygote model (*I*^2^ =55.1%, *P*_heterogneity_<0.001), dominant model (*I*^2^ =46.4%, *P*_heterogneity_<0.001), recessive model (*I*^2^ =60.9%, *P*_heterogneity_<0.001), and allele comparison model (*I*^2^ =58.8%, *P*_heterogneity_<0.001), so that random-effects model was used in all genetic models. Overall, significant association was not identified in all genetic models (homozygote model: OR =1.005, 95% CI =0.931–1.084, *P*=0901, Figure 7; heterozygote model: OR =1.009, 95% CI =0.951–1.070, *P*=0.766, Figure 8; dominant model: OR =0.998, 95% CI =0.951–1.047, *P*=0.932, Figure 9; recessive model: OR =1.005, 95% CI =0.946–1.066, *P*=0.880, Figure 10, and allele comparison model: OR =0.999, 95% CI =0.965–1.035, *P*=0.970, Figure 11). Subgroup analysis was performed according to ethnicity. The same result was found, that is, no significant association was detected in all genetic models amongst Caucasians, Asians, and mixed populations. All the results are listed in Table 3.

**Table 3:** Meta-analysis on the association between *miR-146a* rs2910164 polymorphism and overall cancer risk

Variables	Study number	Statistic model	Test of heterogeneity		Test of association		Publication bias	
			*P*	*I*^2^	OR (95% CI)	*P*	*P*_Begg’s_	*P*_Egger’s_
**Homozygote model**								
***Total***	89	Random	<0.001	57.1	1.005 (0.931–1.084)	0.901	0.568	0.889
***Ethnicity***								
Caucasian	28	Random	0.004	46.9	0.919 (0.716–1.180)	0.756		
Asian	60	Random	<0.001	61.4	0.995 (0.915–1.083)	0.913		
Mixed population	1	Random	-	-	1.01 (0.711–1.435)	0.956		
***Source of control***								
Population-based study	29	Random	<0.001	54.6	1.134 (0.972–1.323)	0.109		
Hospital-based study	60	Random	<0.001	55.4	0.960 (0.882–1.045)	0.347		
**Heterozygote model**								
***Total***	89	Random	<0.001	55.1	1.009 (0.951–1.070)	0.766	0.918	0.836
***Ethnicity***								
Caucasian	28	Random	0.01	42.7	1.072 (0.902–1.273)	0.430		
Asian	60	Random	<0.001	59.3	0.994 (0.934–1.057)	0.839		
Mixed population	1	Random	-	-	0.957 (0.667–1.373)	0.812		
***Source of control***								
Population-based study	29	Random	<0.001	72.9	1.013 (0.863–1.187)	0.878		
Hospital-based study	60	Random	0.005	35	0.997 (0.944–1.052)	0.906		
**Dominant model**								
***Total***	89	Random	<0.001	46.4	0.998 (0.951–1.047)	0.932	0.632	0.349
***Ethnicity***								
Caucasian	28	Random	0.003	48	1.012 (0.929–1.104)	0.781		
Asian	60	Random	<0.001	46.9	0.989 (0.932–1.051)	0.731		
Mixed population	1	Random	-	-	1.048 (0.887–1.240)	0.580		
***Source of control***								
Population-based study	29	Random	0.034	35.1	1.083 (0.983–1.168)	0.420		
Hospital-based study	60	Random	<0.001	46.7	0.957 (0.903–1.015)	0.143		
**Recessive model**								
***Total***	89	Random	<0.001	60.9	1.005 (0.946–1.066)	0.880	0.975	0.817
***Ethnicity***								
Caucasian	28	Random	0.034	35.1	1.083 (1.003–1.168)	0.467		
Asian	60	Random	<0.001	46.7	0.957 (0.903–1.015)	0.743		
Mixed population	1	Random	-	-	0.989 (0.701–1.396)	0.951		
***Source of control***								
Population-based study	29	Random	<0.001	72.3	1.041 (0.895–1.210)	0.605		
Hospital-based study	60	Random	<0.001	50.3	0.986 (0.929–1.046)	0.643		
**Allele comparison model**								
***Total***	89	Random	<0.001	60.8	0.999 (0.965–1.035)	0.970	0.790	0.757
***Ethnicity***								
Caucasian	28	Random	0.002	49.8	1.022 (0.954–1.095)	0.542		
Asian	60	Random	<0.001	65.1	0.991 (0.950–1.032)	0.655		
Mixed population	1	Random	–	–	1.030 (0.899–1.181)	0.670		
***Source of control***								
Population-based study	29	Random	<0.001	57.7	1.053 (0.988–1.122)	0.112		
Hospital-based study	60	Random	<0.001	60.1	0.977 (0.938–1.017)	0.252		

### Publication bias

Egger’s test and Begg’s test were used to investigate the publication bias in the literature in all the genetic models. No publication bias was detected by Begg’s and Egger’s tests. The shapes of the funnel plots (not shown) did not identify obvious asymmetry in any of the comparison models, and plot symmetries are evidenced by *P*-values greater than 0.05. Accordingly, no publication bias was evident in the meta-analysis (Tables 2 and 3).

### Sensitivity analysis

We performed sensitivity analysis by sequential omission of individual studies, and the results showed that the significance of the pooled ORs for *miR-146a* rs2910164 polymorphism was not excessively influenced, suggesting the stability and reliability of the results in the present meta-analysis (not shown).

## Discussion

It is well known that genetic mutations are responsible for cancer occurrence [104]. SNPs, as the most common genetic sequence variation, could affect the function of a series of miRNAs by altering the formation of the primary transcript, miRNA maturation, or miRNA–mRNA interactions [105,106]. Thus, genetic susceptibility to cancer, particularly from SNPs, has been a research focus in the scientific community. Previously, variations of the pre-*miR-146a* C/G gene have drawn increasing attention in cancer etiologies, and altered expression levels have been observed in inflammatory diseases as well as in cancers [107,108]. The results of the present meta-analysis confirm that *miR-146a* C/G polymorphism is associated with HNC risk. This risk is significant amongst the individuals with a dominant genotype model. In the stratified analysis by ethnicity, significant analysis was detected amongst Asians under homozygote and dominant model, while no association was found amongst Caucasians under all genetic models. Furthermore, significant association was found in population-based studies under homozygote, dominant, recessive, and allele comparison models. However, no significant association was detected in hospital-based studies under all genetic models. Moreover, no significant association was found between this gene polymorphism and overall cancer risk. Furthermore, in the stratified analyses by ethnicity and source of control, no significant association was detected in the subgroup analyses of source of control.

To the best of our knowledge, the present study is the first and most comprehensive one to date to assess the relationship between *miR-146a* C/G polymorphism and HNC risk, and the most comprehensive one to date to explore the association between this gene polymorphism and overall cancer risk. Nevertheless, our meta-analysis also has some limitations common to these types of studies. First, relatively large heterogeneity was observed across all the studies involved despite the use of strict criteria for study inclusion and precise data extraction. So, we performed subgroup analyses to explore the possible source of heterogeneity. Second, the majority of subjects included in this meta-analysis were mainly Caucasians and Asians. Thus, the inherent genetic and geographic differences require more data from different ethnic group to increase the statistical power. Third, the low sample size in some of the included studies likely influences the statistical power for evaluating the association between the *miR-146a* C/G polymorphism and HNC risk, especially in subgroup analyses. Fourth, lack of original data from the reviewed studies limited our further evaluation of potential interactions, considering that gene-to-gene and gene-to-environment interactions might modulate cancer risk. As a result, a more precise analysis stratified by variable host factors could be performed. Last, although the results for publication bias were not statistically significant, publication bias may still exist, because only published studies were included in this meta-analysis.

In conclusion, the meta-analysis presented here indicates that *miR-146a* C/G polymorphism more is likely contribute to the susceptibility to HNC, and overall cancer risk. Further well-designed studies with large sample size are needed to confirm these findings.

## Abbreviations

CI: confidence interval
HNC: head and neck cancer
HWE: Hardy–Weinberg equilibrium
OR: odds ratio
SNP: single nucleotide polymorphism
